# MRI-Based Prediction of Meniscal Tear Repairability Demonstrates Limited Accuracy and Reliability

**DOI:** 10.3390/jcm14124160

**Published:** 2025-06-11

**Authors:** Christopher T. Holland, Shannon Tse, Cyrus P. Bateni, Dillon Chen, Cassandra A. Lee

**Affiliations:** 1 Department of Orthopaedic Surgery and Biomedical Engineering, Campbell Clinic Orthopaedics, University of Tennessee Health Science Center, Memphis, TN 38104, USA; cholland@campbellclinic.com; 2 Department of Orthopaedic Surgery, University of California Davis, 3301 C Street, Suite 1600, Sacramento, CA 95816, USA; sstse@ucdavis.edu; 3 Department of Radiology, University of California Davis, 4860 Y Street, Suite 3100, Sacramento, CA 95817, USA; cpbateni@ucdavis.edu (C.P.B.); dcychen@ucdavis.edu (D.C.)

**Keywords:** meniscus, meniscal repair, MRI, repairability, reliability

## Abstract

**Background:** While magnetic resonance imaging (MRI) is commonly used to identify meniscal tears, intraoperative assessment typically dictates repairability. This study evaluated whether a simplified MRI-based scoring system could reliably predict meniscal repair versus meniscectomy. **Methods:** Patients who underwent meniscectomy or meniscal repair between 2010 and 2018 were retrospectively identified. Preoperative MRIs were independently reviewed in a blinded fashion by two radiologists and one orthopedic sports surgeon. Reviewers scored images based on four arthroscopic criteria for tear repairability, with one point awarded for each of the following criteria—(1) proximity within 4 mm of the meniscosynovial junction, (2) length > 10 mm, (3) presence of intact inner meniscal segment, and (4) >50% meniscal thickness. Tears scoring four points were considered repairable. Accuracy, sensitivity, and positive and negative predictive values were calculated against the actual procedure performed. Inter- and intraobserver reliability were evaluated using kappa statistics. The predictive performance of each individual criterion was also analyzed. **Results:** A total of 202 meniscal tears were included (134 meniscectomies and 68 repairs). Reviewer accuracy in predicting repairability ranged from 48% to 76%. Intraobserver reliability was moderate to substantial (κ = 0.42–0.66), whereas interobserver reliability was poor to moderate (pairwise κ = 0.07–0.43; Fleiss’ κ = 0.11). Analysis of individual MRI criteria demonstrated limited predictive value, with most criteria achieving less than 50% accuracy across reviewers. **Conclusions:** MRI-based prediction of meniscal repairability using arthroscopic criteria demonstrated limited accuracy and poor interobserver reliability. Overall predictive reliability remains insufficient for clinical decision-making. Further investigation, integrating advanced imaging techniques and artificial intelligence, may improve the preoperative assessment of meniscal repairability.

## 1. Introduction

The meniscus is the most commonly injured structure in the knee joint and remains the leading reason for knee arthroscopy [1]. Meniscal tears are usually treated with either partial meniscectomy or meniscal repair. Magnetic resonance imaging (MRI) is the gold standard for diagnosing a meniscal tear [2], however, its role has typically been limited to confirming the presence of a tear, with the final decision to repair or resect made intraoperatively by the surgeon.

The decision to perform a meniscal repair is a subjective evaluation influenced by multiple factors, including tear morphology, location, patient age, level of activity, as well as the surgeon’s individual experience [3]. Patients who are candidates for repair are counseled that the tear will be directly assessed intraoperatively, and the final determination between resection versus repair will be made at that time. This subjectivity leads to variability in treatment decisions, even when preoperative imaging appears favorable.

Historically, meniscal repair has been guided by several well-accepted arthroscopic criteria, rooted in vascular anatomy and tear biomechanics. Tears located in the peripheral third of the meniscus, also known as the “red–red zone”, are considered the most amenable to repair due to their robust vascular supply, while those in the middle third (“red–white zone”) may also be repairable depending on patient and tear characteristics. Conversely, tears in the avascular inner third (“white–white zone”) are less likely to heal and are typically resected [4,5]. Longitudinal tears greater than 10 mm in length, with an intact inner rim and at least 50% remaining meniscal thickness, are also considered favorable for repair. These principles form the basis of commonly used intraoperative repairability criteria.

Meniscal repair has been shown to result in favorable clinical and functional outcomes [6,7], with some studies suggesting it should be preferred over meniscectomy when feasible [8,9]. In recent years, there has been a growing emphasis on preserving meniscal tissue, driven by increasing evidence linking meniscectomy to higher rates of osteoarthritis [3,10]. However, repair procedures are more time-consuming, technically complex, and require more restrictive and prolonged postoperative rehabilitation [11]. Regardless of the procedure performed, meniscal injuries carry a substantial impact on short and long-term patient outcomes, including return to activity, quality of life, and the risk of osteoarthritis development [8].

Accurately predicting meniscal repairability preoperatively remains a challenge. Several studies, primarily with smaller sample sizes, have demonstrated significant variation in the ability to predict the repairability of meniscal tears. Reported inter- and intraobserver reliability measures range widely from poor to excellent, and diagnostic performance metrics such as sensitivity, specificity, predictive values, and overall accuracy have similarly shown inconsistent outcomes [12,13,14,15,16]. This inconsistency reflects the difficulty of applying MRI findings uniformly to predict surgical decision-making across different tear types and surgeon practices. Nevertheless, developing a reliable, simplified MRI-based approach for assessing repairability could still provide clinical value by offering a consistent framework for evaluating tear characteristics. Such a tool may support more informed and objective preoperative discussions, improve patient counseling, and reduce variability in surgical planning, particularly in settings with less experienced surgeons or in multidisciplinary practices. Even if variability in final intraoperative decisions persists, a preoperative predictive system could help align expectations, improve documentation, and guide future research efforts to refine meniscal treatment.

The aim of this study was to evaluate whether a simple four-criterion MRI-based scoring system could reliably predict meniscal repairability. This investigation is distinguished by its relatively large cohort, the inclusion of both radiologists and an orthopaedic surgeon, and a dedicated sub-analysis of each criterion of the scoring system, as well as patients with concomitant anterior cruciate ligament (ACL) injuries.

## 2. Materials and Methods

Following Institutional Review Board approval, a retrospective review of patients who underwent meniscectomy or meniscal repair at a single academic center between 1 January 2010 and 1 September 2018 was conducted. Cases were identified using Current Procedural Terminology (CPT) codes 29880, 29881, 29882, and 29883, which pertain to arthroscopic meniscus procedures. Patients aged 16 and over with available preoperative knee MRI were included. Exclusion criteria included absence of preoperative MRI, MRI performed at an outside institution, or a time interval greater than 3 months between MRI and surgery. All MRIs were performed using 1.5 Tesla (T) or 3T MRI scanners (General Electric Signa HDxt and Optima GE450w, Milwaukee, WI, USA) and included axial, coronal, and sagittal proton density and fat saturation sequences. All surgeries were performed by board-certified orthopedic sports medicine surgeons. As patients were treated over a prolonged period at a large academic center, variability in surgical decision-making among providers and the use of both 1.5 T and 3 T MRI scanners were unavoidable in this retrospective study.

**Figure 1:**
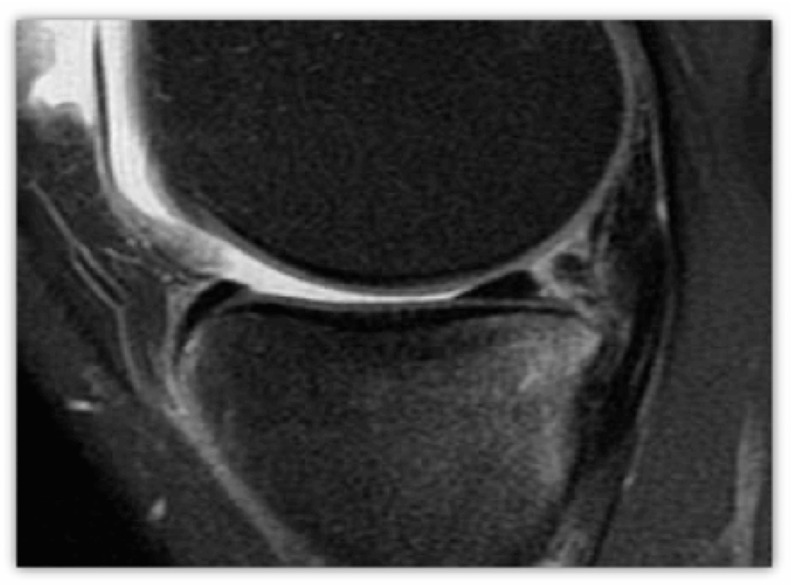
Example of a true positive, found to be repairable by all 3 reviewers (score = 4) and repaired intraoperatively. This sagittal proton density fat saturation MRI of the knee shows a longitudinal tear through the posterior horn of the medial meniscus, with a vertically oriented high-signal cleft at its posterior periphery and bone marrow edema within the posterior medial tibial plateau.

**Figure 2:**
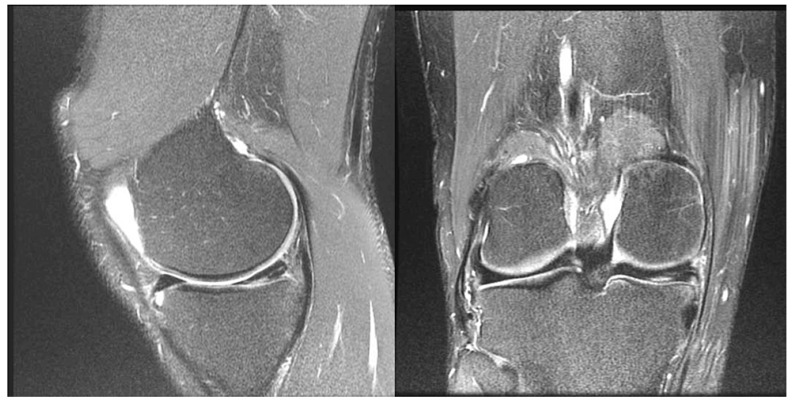
Example of a true negative. Predicted to be non-repairable by reviewers 2 and 3, but repairable by reviewer 1. Found non-repairable during arthroscopy. Sagittal (**left**) and coronal (**right**) proton density fat saturation MRI with a horizontal increased signal at the undersurface of the posterior horn of the medial meniscus from meniscal tearing. The tear is of less than 50% of the meniscal thickness.

Patient demographic details, including age and sex, and the surgical treatment (meniscectomy or meniscal repair) were recorded. A single-blind review of preoperative MRI imaging was performed by two board-certified musculoskeletal radiologists (DC and CPB, with 19 and 8 years of experience, respectively), and one fellowship-trained sports orthopedic surgeon (CAL, 11 years of experience). CTH and ST did not participate in the radiological assessments. Reviewers were instructed to evaluate intraoperative repairability using four established arthroscopic criteria [12,13,14]: (1) proximity within 4 mm of the meniscalsynovial junction, (2) >10 mm in length, (3) an intact inner meniscal segment, and (4) >50% of meniscal thickness. These criteria were selected based on well-established intraoperative repairability principles described in prior studies [12,13,14], and represent commonly used decision-making factors during surgery. One point was given for each criterion (0 to 4). Tears scoring 4 were predicted to be repairable (Figure 1), and those scoring <4 as irreparable (Figure 2). All patients were anonymized, and reviewers were blinded to clinical information. Reviewers were able to view all image sequences and manipulate the images as necessary. Each reviewer assessed all images twice, with six weeks between the two rounds of scoring. Prior to independent assessment, all reviewers participated in a calibration session where a subset of cases was reviewed together and each criterion was discussed. A standardized reference guide was provided to ensure consistent interpretation throughout the grading process. Additionally, reviewers classified tear type based on the International Society of Arthroscopy, Knee Surgery and Orthopaedic Sports Medicine (ISAKOS) classification—bucket handle, horizontal, radial, vertical flap, horizontal flap, or complex [17].

The actual surgical procedure performed was used as the reference standard. Sensitivity, specificity, positive (PPV) and negative predictive values (NPV), and accuracy were calculated based on each reviewer’s MRI-based prediction relative to the operative outcome. These metrics were also independently assessed for each of the four individual repairability criteria. Interobserver reliability was calculated using Fleiss’ kappa across all three reviewers and Cohen’s kappa for each reviewer pair across both rounds. Intraobserver reliability was assessed using Cohen’s kappa by comparing each reviewer’s first and second scores. Kappa statistical values were interpreted with the Landis and Koch criteria [18]: slight (<0.2), fair (0.21 to 0.40), moderate (0.41 to 0.60), substantial (0.61 to 0.80), and almost perfect (>0.8). A subgroup analysis was performed for meniscal tears with concomitant ACL tears. Statistical analysis was performed using JMP (Version 16.0. SAS Institute Inc., Cary, NC, USA).

## 3. Results

A total of 202 meniscal tears met the inclusion criteria for final analysis. These tears were identified in 193 separate knees, with 9 having both medial and lateral meniscal tears that were analyzed separately. The mean age of patients was 31.9 years, and 118 (61.1%) were male. Among the 202 tears, 134 (66.3%) underwent meniscectomy and 68 (33.7%) underwent meniscal repair. There were no significant differences in age, sex, or tear location between the meniscectomy and repair groups. Reviewer predictive performance is summarized in Table 1. Overall, predictive accuracy ranged from 48 to 76%, with notable variability in sensitivity (40–75%), specificity (35–91%), and PPV (37–70%) across reviewers (Table 2). NPV was relatively consistent across reviewers, ranging from 73 to 79%.

Interobserver reliability across reviewers ranged from poor to moderate, with pairwise Cohen’s kappa values ranging from 0.07 to 0.43 (Table 3). Overall agreement across all three reviewers was poor, with a Fleiss kappa of 0.11 (Table 2). Intraobserver reliability was moderate to substantial, with Cohen’s kappa values of 0.42, 0.43, and 0.66. Intraobserver percent agreement ranged from 74.8% to 86.6% (Table 2). These findings underscore the inherent challenges in interpreting meniscal repairability on MRI, with variability likely reflecting differences in image interpretation and thresholding between reviewers.

Further analysis of the individual MRI repairability criteria demonstrated limited predictive value, with most reviewers achieving less than 50% accuracy for the criterion (Table 4). While sensitivity was generally high, specificity and PPV remained low. This suggests that while reviewers were able to identify potentially repairable features, these criteria alone were insufficiently specific to reliably differentiate repairable from irreparable tears.

In the subset of 74 tears with concomitant ACL injuries (Table 5), overall reviewer prediction accuracy improved minimally. Intraobserver reliability in this group ranged from fair (Reviewer 2, k = 0.38) to almost perfect (Reviewer 3, k = 0.8). Pairwise interobserver agreement remained poor to moderate: k = 0.09 for Reviewer 1 vs. Reviewer 2 (95% CI: −0.13–0.30, *p* = 0.433), 0.30 for Reviewer 1 vs. Reviewer 3 (95% CI: 0.11–0.49, *p* = 0.002), and 0.42 for Reviewer 2 vs. Reviewer 3 (95% CI: 0.25–0.60, *p* < 0.001). Fleiss’ kappa across all three reviewers was fair overall (k = 0.21). Similar to the overall cohort, Reviewer 3 had the highest overall diagnostic accuracy (76%).

Among the 84 cases (41.6%) where all reviewers agreed on morphology, bucket handle tears were the most common (*n* = 43), followed by 14 horizontal tears, 14 radial tears, 1 vertical flap tear, 5 horizontal flap tears, and 7 complex tears. Among bucket handle tears, 22 (51.2%) were treated with repair. When evaluating only bucket handle tears, Reviewer 3 demonstrated the highest predictive accuracy at 71%, with a sensitivity of 77% and specificity of 64%. Reviewer 2 demonstrated moderate predictive performance, with 62% accuracy, 43% sensitivity, and 83% specificity. Reviewer 1 had the highest sensitivity (95%) but a markedly low specificity (7%), resulting in a lower accuracy of 53% and frequent overprediction of repairability. These findings suggest that certain tear morphologies, such as bucket handle tears, may be more amenable to accurate MRI-based prediction, possibly due to their more recognizable features. However, prediction remained inconsistent across reviewers, underscoring ongoing limitations even in tear subtypes.

## 4. Discussion

In this study, we evaluated whether a simple MRI-based scoring system could reliably predict the repairability of meniscal tears. Despite a cohort of over 200 tears and the inclusion of both radiologists and an orthopaedic surgeon, predictive accuracy remained limited. Reviewer accuracy ranged widely between 48% and 76%, and interobserver reliability remained poor to moderate. These findings reinforce the challenges previously reported in the literature and highlight that surgical decision-making regarding meniscal treatment remains complex and subjective.

Despite advances in imaging technology and a structured scoring approach, predictive performance has remained poor to moderate across multiple studies [12,14,15,16]. The average overall accuracy of 66% we found in our study was similar to that of Matava et al. (74%) [12] and Bernthal et al. (60%) [14]. Bernthal et al. also reported that despite using similar criteria, examiners agreed only 38% of cases [14]. Additionally, Strawbridge et al. found that 3 T MRI offered no significant improvement in predictive performance compared to 1.5 T scanners [16]. Our study allowed reviewers to use all MRI sequences for their grading and included images derived from both 1.5 T and 3 T scanners. While no formal subgroup analysis was performed based on scanner strength in our study, and though this may introduce variability into this study, it more accurately reflects clinical practice, where both 1.5 and 3 T MRI scanners are used and a variety of sequences are used in interpretation.

Analysis of individual MRI-based criteria similarly demonstrated poor predictive value. While these features have been proposed as markers of repairability, our detailed subanalysis showed that the accuracy for each criterion remained below 50% for most reviewers. Bernthal et al. reported that an intact inner meniscal segment was the most predictive individual criterion; however, it only reached statistical significance in one of two reviewers [14]. Van der Wal et al. investigated whether specific MRI criteria, including peripheral rim width, tear length, and homogeneity of meniscal tissue, could reliably predict repairability of longitudinal full-thickness medial or lateral meniscal tears in their 63-patient cohort [13]. While they reported moderate to excellent intraobserver agreement for measuring tear and rim width, and moderate overall interobserver agreement, these MRI measurements did not correlate with whether the meniscal repair was successful. These findings mirror those of our study, reinforcing that even when tear characteristics can be reliably measured on MRI, they do not consistently predict repairability. Our study also expanded beyond longitudinal tears to include a broader variety of tear morphologies, which likely contributed to the lower predictive accuracy compared to Van der Wal et al. [13].

Felisaz et al. evaluated the ability of MRI to predict meniscal repairability based solely on the distance of the tear from the meniscosynovial junction, reporting an accuracy of 83%, sensitivity of 85%, specificity of 79%, and a PPV of 86% across 79 patients [19]. They also demonstrated almost excellent interrater reliability. However, when we analyzed the same variable within our larger and more heterogeneous cohort, mean reviewer accuracy was lower at 66%. Differences in tear morphology, broader patient demographics, and surgical management by four different orthopedic surgeons in our study, compared to a single surgeon in the study by Felisaz et al., likely contributed to this discrepancy and may better reflect the variability encountered in routine clinical practice.

Kumaraswamy et al. proposed a complex scoring model, the “Ortho One PROMT”, incorporating age, chronicity of tear, Kellgren and Lawrence radiographic grade, zone of the tear, and pattern of the tear for predicting meniscal repairability [20]. They reported high sensitivity and specificity for medial meniscal tears (90.9% sensitivity and 93.2% specificity for medial repair; 93.2% sensitivity and 90.9% specificity for medial meniscectomy), and lower predictive accuracy for lateral tears (69.2% sensitivity for repair and 78.8% sensitivity for meniscectomy). Although these results appear promising, methodological concerns have been raised, including limited MRI sensitivity for tear detection, lack of standardized imaging protocols, unclear sample selection, and inconsistencies in reported data [21]. Additionally, the complexity of their scoring system, requiring multiple clinical and imaging parameters, may limit its practicality in routine practice.

In a survey study of orthopedic surgeons evaluating clinical profiles of middle-aged patients with symptomatic non-obstructive meniscal tears, surgeons correctly predicted treatment outcomes only 50% of the time, no better than chance, further highlighting the difficulty of accurately predicting outcomes for patients with meniscal tears [22]. Notably, experienced knee surgeons performed no better than general orthopedic surgeons. These findings emphasize that even with clinical experience, reliably predicting the optimal management of meniscal tears remains challenging and reinforce the need for caution when using preoperative imaging or clinical features alone to guide surgical decision-making.

Given the persistent limitations of conventional MRI interpretation and manual grading systems, particularly subjectivity, variability between raters, and limited predictive accuracy demonstrated in our study, future research may focus on integrating advanced imaging modalities and artificial intelligence (AI) to improve predictive accuracy. For example, deep learning models trained on large, annotated datasets may reduce interobserver variability by providing consistent, automated assessments of tear characteristics, while simultaneously identifying subtle imaging features not easily recognized by human reviewers. AI algorithms could also integrate multiple clinical and imaging variables to generate individualized repairability predictions, improving preoperative planning and patient counseling. Ultra-high field 7 T MRI scanners have shown superior diagnostic performance in detecting cartilage and meniscal damage [23], which may enhance the preoperative assessment of repair-relevant features such as rim integrity and proximity to the vascular zone. Deep learning models have demonstrated promising results in MRI-based knee pathology detection and classification, with reported area under the receiver operating characteristic curve of 0.847 to 0.992 and diagnostic accuracies up to 90% for meniscal tears [24,25,26]. AI-assisted grading has also been shown to improve interobserver agreement in knee MRI interpretation [27]. The successful application of AI by Salman et al.’s study in predicting total knee arthroplasty implant sizes with up to 99% accuracy [28] further highlights the potential of these technologies in refining meniscal treatment strategies in the future. Lastly, it is possible that imaging features beyond the four primary intraoperative criteria assessed in this study may exist and could further enhance the prediction of meniscal repairability. With advancements in surgical techniques, instrumentation, and biologic augmentation, there has been a growing trend toward more aggressive meniscal preservation, even in tears previously considered irreparable [10]. This evolving philosophy highlights an important distinction between predicting whether a tear will be repaired versus whether it will ultimately heal. While our study focused on intraoperative repairability, future research should differentiate between repairability and healing potential, as the latter may be better captured through biological and vascular features visible on advanced MRI and explore predictive models for true healing outcomes.

Our study is the largest to date to include both orthopedic surgeons and radiologists concurrently evaluating preoperative MRIs for meniscal repairability, reflecting the multidisciplinary nature of real-world clinical practice. The study by Misir et al. included 223 patients but had only orthopaedic reviewers and similarly found high variability in predicting tear repairability [15]. Additionally, our cohort of 202 tears was evaluated using both 1.5 T and 3 T MRI scanners, enhancing the generalizability of our findings to diverse clinical settings. We also performed a focused subanalysis of patients with concomitant ACL injuries, acknowledging that tear patterns and reparability considerations may differ in this population. Furthermore, we separately analyzed each of the four arthroscopic repairability criteria to assess their individual predictive performance, providing a more granular evaluation.

However, several limitations should be noted. First, the retrospective nature of this study introduces inherent selection and information biases and precludes standardized intraoperative documentation of the arthroscopic repairability criteria. Treatment decisions were left at the surgeon’s discretion. Surgical interventions were performed by four different surgeons, each with potentially differing thresholds for repair versus meniscectomy, which introduces inter-surgeon heterogeneity in treatment classification and limits standardization. However, such differences are typical in clinical practice and may reflect regional or institutional variation, which enhances the real-world applicability of our findings. Third, both 1.5 T and 3 T MRI scanners were used without a standardized imaging protocol, potentially introducing variability in tear characterization. Nonetheless, this variability aligns with the spectrum of MRI equipment and protocols used across healthcare systems.

To minimize bias from clinical decision-making variability, an objective four-criterion scoring system was applied to all cases, and MRIs were evaluated independently by experienced reviewers blinded to clinical and surgical outcomes. Additionally, shifts in surgical philosophy towards favoring meniscus preservation may have influenced treatment decisions during the study period. While this heterogeneity limits strict reproducibility, it enhances the generalizability of our findings. Furthermore, patient preferences regarding repair versus resection were not systematically assessed and may have influenced surgical choices. While we did not include patient-specific factors such as age in our MRI-based scoring system, this reflects our study’s focus on evaluating the diagnostic value of structural MRI findings alone. Though age may influence surgeon decision-making in select cases, it is increasingly recognized that age alone is not a strict contraindication to meniscal repair. Factors such as cartilage quality, chronicity, and patient goals are likely more relevant to clinical outcomes. These are important variables to consider in future comprehensive predictive models that combine imaging with clinical data.

## 5. Conclusions

Our findings reaffirm the limitations of MRI as a standalone tool for reliably predicting meniscal repairability, even when using simple, pragmatic criteria that are routinely used intraoperatively. Despite ongoing technological improvements, predictive performance remains modest and highly dependent on clinical judgment. Given these limitations, surgeons should be cautious in relying solely on MRI to guide preoperative decisions regarding meniscal repair and instead continue to prioritize intraoperative assessment alongside patient-specific factors. Future research should focus on enhancing predictive modeling through advanced imaging modalities and the application of AI techniques. As treatment strategies for meniscal injuries continue to evolve, integrating clinical assessment, patient-specific factors, and advanced imaging analytics will be critical to improving individualized patient care and optimizing surgical decision-making.

## Figures and Tables

**Table 1:** Reviewer predictions of meniscal repairability compared to the actual procedure.

	Reviewer 1		Reviewer 2		Reviewer 3	
Actual Procedure	Predicted Repair	Predicted Meniscectomy	Predicted Repair	Predicted Meniscectomy	Predicted Repair	Predicted Meniscectomy
Meniscectomy (*n* = 134)	88 (65.7%)	46 (34.3%)	12 (9.0%)	122 (91.0%)	18 (13.4%)	116 (86.6%)
Repair (*n* = 68)	52 (76.5%)	16 (23.5%)	28 (41.2%)	40 (58.8%)	37 (54.4%)	31 (45.6%)

**Table 2:** Overall interobserver reliability, intraobserver reliability, and diagnostic performance in predicting repairability.

Reviewer	Accuracy (%)	NPV (%)	PPV (%)	Specificity (%)	Sensitivity (%)	Intraobserver Percent Agreement (%)	Intraobserver Cohen’s Kappa (SE; 95% CI)	Interobserver Fleiss’ Kappa (SE; 95% CI)
Reviewer 1	48	73	37	35	75	74.8	0.42 (0.076; 0.27 to 0.57)	0.11 (0.066; −0.02 to 0.24)
Reviewer 2	74	75	70	91	40	81.2	0.43 (0.075; 0.28 to 0.58)	
Reviewer 3	76	79	68	87	54	86.6	0.66 (0.058; 0.54 to 0.77)	
Mean	66	76	58	71	53	80.7	0.50 (0.070)	

Using combined round 1 and 2 scoring. SE, standard error; CI, confidence interval; PPV, positive predictive value; NPV, negative predictive value.

**Table 3:** Pairwise interobserver reliability with Cohen’s kappa.

Comparison	*p*-Value	95% CI	SE	Interobserver Kappa
Reviewer 1 vs. Reviewer 2	0.274	−0.06 to 0.21	0.068	0.07
Reviewer 1 vs. Reviewer 3	0.004	0.06 to 0.31	0.064	0.18
Reviewer 2 vs. Reviewer 3	<0.001	0.32 to 0.53	0.053	0.43

Using combined round 1 and 2 scoring. SE, standard error; CI, confidence interval.

**Table 4:** Diagnostic performance via individual repairability criterion.

Reviewer	Accuracy (%)	NPV (%)	PPV (%)	Specificity (%)	Sensitivity (%)
**Criteria 1: Proximity within 4 mm of the meniscosynovial junction**					
Reviewer 1	41	90	36	13	97
Reviewer 2	75	76	70	90	45
Reviewer 3	59	84	44	46	83
Mean	58	83	50	50	75
**Criteria 2: >10 mm in length**					
Reviewer 1	40	66	34	19	81
Reviewer 2	46	81	37	25	88
Reviewer 3	49	71	36	40	67
Mean	45	73	36	28	79
**Criteria 3: Intact inner meniscal segment**					
Reviewer 1	40	86	35	12	96
Reviewer 2	48	76	37	31	81
Reviewer 3	72	90	55	65	86
Mean	58	84	42	36	88
**Criteria 4: >50% of meniscal thickness**					
Reviewer 1	35	79	34	4	98
Reviewer 2	38	91	35	8	99
Reviewer 3	57	85	43	42	86
Mean	43	85	37	18	94

Using combined round 1 and 2 scoring. PPV, positive predictive value; NPV, negative predictive value.

**Table 5:** Subgroup Analysis of Meniscal Tears with Concurrent ACL Repair.

Reviewer	Accuracy (%)	NPV (%)	PPV (%)	Specificity (%)	Sensitivity (%)	Intraobserver Cohen’s Kappa (SE; 95% CI)	Interobserver Fleiss’ Kappa (SE; 95% CI)
Reviewer 1	59	64	56	39	78	0.4 (0.127; 0.15 to 0.65)	0.21 (0.103; 0.01 to 0.41)
Reviewer 2	66	61	79	88	45	0.38 (0.129; 0.13 to 0.63)	
Reviewer 3	76	72	83	87	66	0.8 (0.073; 0.66 to 0.95)	
Mean	67	66	73	71	63	0.53 (0.110)	

Using combined round 1 and 2 scoring. SE, standard error; CI, confidence interval; PPV, positive predictive value; NPV, negative predictive value.

## Data Availability

The raw data supporting the conclusions of this article will be made available by the authors on request.
